# RE: Risk of HBV Reactivation in HBsAg Negative and Anti-HBc IgG Positive Patients Receiving Biologic Therapy

**DOI:** 10.5152/tjg.2023.237002

**Published:** 2023-03-01

**Authors:** İlkay Ergenç, Haluk Tarık Kani, Murat Karabacak, Elif Cömert Özer, Shahin Mehdiyev, Fuad Jafarov, Kerem Yiğit Abacar, Seda Kutluğ Ağaçkıran, Gizem Sevik, Rahmi Aslan, Fatma Alibaz Öner, Nevsun İnanç, Mehmet Pamir Atagündüz, Dilek Seçkin, Yeşim Özen Alahdab, Tülin Ergun, Haner Direskeneli, Özlen Atuğ

**Affiliations:** 1 Department of Internal Medicine, Division of Gastroenterology, Marmara University, School of Medicine, Istanbul, Turkey; 2 Department of Internal Medicine, Division of Rheumatology, Marmara University, School of Medicine, Istanbul, Turkey; 3 Department of Dermatology, Marmara University, School of Medicine, Istanbul, Turkey

We thank Dr. Sargın^1^ for her comments regarding our recently published article^2^ indicating the very low risk of hepatitis B reactivation in hepatitis B surface antigen (HBsAg)-negative and hepatitis B core immunoglobulin G antibody (anti-HBc IgG)-positive patients under biologic treatment.

She raised concerns that the absence of follow-up hepatitis B virus (HBV) DNA levels in a small group of patients may cause an underestimation of the hepatitis B reactivation rate in our study. We agree with the author about the importance of HBV DNA monitoring at least 3-4 times in the first year.^2^ As we discussed in the manuscript, this was one of the main limitations of our real-life retrospective study. Nevertheless, our study population was one of the largest samples of HBsAg-negative and anti-HBc IgG-positive patients exposed to several biologic agents. Despite this limitation, we believe that it makes an important contribution to the literature.

In the letter, the author emphasized that three-quarters of the study population were not screened for the presence of HBV DNA prior to biologic exposure. She also pointed out the probability of occult hepatitis B infection in our population. Occult HBV infection is a challenging clinical entity defined as the presence of replication-competent viral DNA in the liver of HBsAg negative individuals with or without detectable HBV DNA in the serum.^3^ Therefore, we cannot rule out the possibility of occult HBV infection with HBV DNA testing. The EASL 2017 clinical practice guidelines on managing HBV infection indicate that the HBsAg-negative and anti-HBc IgG-positive phase (phase 5) of chronic HBV infection may also be named “occult HBV infection.”^4^ It is unclear whether the presence of low levels of HBV DNA in the serum increases the risk of HBV reactivation under biological treatment. We believe that the absence of baseline HBV DNA measurements does not weaken the study’s results. If subjects with detectable HBV DNA cases were diagnosed and excluded from the study, the reactivation rate would be more likely to decrease than increase.

Finally, we agree with the author that in clinical practice, HBV DNA should be tested before biological exposure in these patients. Pre-emptive therapy seems the safest and most cost-effective way to prevent HBV reactivation in HBsAg-negative and anti-HBc IgG-positive patients under biologic treatment.
